# Alterations in the gut microbiota of Chinese patients following *Helicobacter pylori* eradication with bismuth-based quadruple therapy

**DOI:** 10.3389/fmicb.2025.1713477

**Published:** 2025-12-10

**Authors:** Xiao-Juan Chen, Ming-Ye Hu, En-Dian Zheng, Ju-Yi Pan

**Affiliations:** Department of Gastroenterology, Wenzhou People’s Hospital, Wenzhou Third Clinical Institute Affiliated to Wenzhou Medical University, Wenzhou, Zhejiang, China

**Keywords:** diversity, eradication, gut microbiota, *Helicobacter pylo*ri, microbiota composition, relative abundance

## Abstract

**Objective:**

The aim of this study is to investigate the changes in gut microbiota before and after *Helicobacter pylori* (*Hp*) eradication, assess the structural distribution of gut colonies in individuals infected with *Hp*, and examine the alterations in gut microbiota following *Hp* eradication.

**Methods:**

Patients diagnosed with *Hp* infection between June 1, 2021, and December 31, 2021, were included in the study. A total of 26 patients who underwent standard quadruple first-line therapy for *Hp* eradication were prospectively enrolled. Fecal samples were collected 1 day before treatment and 6 weeks after treatment. The gut microbiota was sequenced using the 16S rRNA next-generation gene sequencer to assess changes in microbiota composition ratios and diversity, before and after treatment.

**Results:**

Among the 26 patients with *Hp* infection, aged between 19 and 55 years, 18 had positive results in the ^13^C urea breath test, while 8 were diagnosed through gastroscopic histopathological examination. Changes in gut microbiota diversity were observed before and after *Hp* eradication. At 56 days post-treatment, alpha diversity changes were not significant, whereas beta diversity changes differed in the gut microbiota. Variations were also noted in the relative abundance and composition ratios of the gut microbiota at the phylum and genus levels before and after *Hp* eradication. Compared to the pre-eradication state, the metabolic pathways of the gut microbiota were less abundant following *Hp* eradication.

**Conclusion:**

Significant changes were observed in the beta diversity of the gut microbiota, the relative abundance at the phylum and genus levels, and metabolic pathways within a short period following *Hp* eradication.

## 1 Introduction

The discovery of *H. pylori* revolutionized gastroenterology. Before 1979, the human stomach was thought sterile, until Robin Warren observed spiral bacteria in gastritis patients’ gastric biopsies (Marshall and Warren, 1984). Nearly half of the world’s population is infected by *Helicobacter pylori* (*Hp*) through fecal-oral transmission route and *in vivo* colonization, with an infection rate of approximately 50% in China (Hooi et al., 2017). *H. pylori* is introduced as “infecting” half of the world’s population, it is classified as a Group 1 carcinogen. There is a significant discordance in the literature whether *Hp* is a beneficial commensal when not causing acute “infection.” *Hp* infection can lead to gastroduodenal diseases, including acute active gastritis (90% positive for *Hp*) (You et al., 2000), peptic ulcer disease (61.1% duodenal ulcers, and 58.6% gastric ulcers, positive for *Hp*) (Morrison and Preston, 2016), gastric cancer (1.5–2.0% infected individuals progress to gastric cancer, and *Hp* is classified as a Group 1 carcinogen) (IARC Working Group on the Evaluation of Carcinogenic Risks to Humans, 1994), and mucosa-associated lymphoid tissue (is connected to *H. pylori* about two out of three) (Raderer and Kiesewetter, 2020). The methods for detecting and diagnosing *Hp* include gastric mucosal biopsy staining, polymerase chain reaction (PCR) detection of *Hp* DNA, rapid urease test (RUT) or bacterial culture, urea breath test (UBT) or fecal antigen test (SAT) (Sugano et al., 2015). *Hp* infection typically persists for a lifetime unless treated with antibiotics or spontaneously cleared due to extensive gastric atrophy and metaplasia resulting from long-term infection (Treiber et al., 2003; Mejías-Luque and Gerhard, 2020). Most *Hp* infections occur in early childhood, with fewer cases in adults.

Previous studies, such as a prospective study by Uemura et al. (2001) have demonstrated that the risk of gastric cancer in Hp-infected patients is approximately 2.9% over a mean follow-up period of 7.8 years (Uemura et al., 2001). Additionally, comprehensive evidence from multiple studies suggests that the lifetime risk of gastric cancer in *H. pylori*-infected patients is generally in the range of 1–3% (Wiklund et al., 2025), with some estimates falling within the 1.5–2.0% range depending on various factors (Uemura et al., 2001). Epidemiologic findings have confirmed an association between *Hp* infection and an increased risk of colorectal cancer (pooled OR = 1.39–1.42, 95% CI 1.18–1.46), with this correlation varying slightly by geographic region and economic development level (Coelho and Coelho, 2021; Dong and Liang, 2015; Ishaq and Nunn, 2015). *Hp* eradication can cure acute active peptic ulcer disease (PUD) and reduce the incidence of gastric cancer.

The human gut microbiota plays a key role in the body’s immune, physiological, and metabolic systems (Kamada et al., 2013; Xu et al., 2024). Current first-line bismuth-based quadruple therapy for *H. pylori* has replaced obsolete triple therapy due to high global clarithromycin resistance (> 20%, 30–40% in China) (Coelho and Coelho, 2021), yet treatment faces major challenges including widespread antibiotic resistance (e.g., 78% to metronidazole in China) (Chen et al., 2022), GI/systemic side effects, and poor adherence—which, at 65–70% in China, increases failure risk by 3.2-fold (Zhu and Wu, 2019). Bismuth-based quadruple therapy containing two antibiotics cannot selectively target harmful microorganisms (e.g., H. pylori), thereby causing a negative impact on beneficial microbes within the gut profile. However, the specific effects of the *H. pylori* eradication regimen on the gut microbiota remain inadequately characterized, particularly in a longitudinal study design that tracks recovery. Much of the existing literature on antibiotics and the gut microbiota focuses on general courses of antibiotics (Fishbein et al., 2023), leaving a gap regarding the precise impact of this specific and commonly prescribed therapeutic regimen. Given the critical role of the gut microbiota in health, understanding the consequences of its perturbation by *H. pylori* therapy is of paramount clinical importance. Furthermore, beyond merely describing taxonomic shifts, this study provides distinct advancements by employing PICRUSt2 to infer the potential functional consequences of the observed microbiota alterations, thereby offering an in-depth functional inference linking microbiota changes to host physiology. Therefore, the primary aim of this study was to longitudinally investigate the alterations in the composition and diversity of the gut microbiota in patients with *H. pylori* infection before and after bismuth-based quadruple therapy. We hypothesized that this eradication therapy would lead to a significant short-term reduction in microbial alpha-diversity and cause distinct shifts in beta-diversity and taxonomic composition, potentially marked by a decrease in beneficial short-chain fatty acids (SCFAs)-producing taxa.

## 2 Materials and methods

### 2.1 General data

#### 2.1.1 Objects

Patients with chronic gastritis who tested positive in the ^13^C urea breath test or were identified as *Hp*-positive based on gastric mucosal histopathology between June 1, 2021, and December 31, 2021, were selected for this study. Written informed consent was provided by all the patients prior to enrollment.

Inclusion criteria: (1) Patients aged between 18 and 65 years; (2) Patients first identified as *Hp*-positive with no history of sterilization. Exclusion Criteria: (1) Pregnant or breastfeeding patients; (2) Patients who received antibiotics within 1 month prior to enrollment; (3) Patients diagnosed with or suspected of having malignant tumors of the upper digestive tract or other severe digestive tract diseases; (4) Patients with a personal history of malignant tumors; (5) Patients known to be allergic to any drugs used in this study; (6) Patients with severe concomitant diseases; (7) Patients with a history of gastrointestinal surgery.

All eligible patients received standard quadruple first-line therapy for *Hp* eradication, consisting of tetracycline tablets (0.5 g, tid), amoxicillin capsules (1.0 g, bid), bismuth potassium citrate capsules (220 mg, bid), and lansoprazole tablets (30 mg, bid), administered for 14 consecutive days. These drugs could not be discontinued or altered without permission. Subsequently, an analysis was conducted to evaluate the changes in the gut microbiota before and after *Hp* eradication.

#### 2.1.2 Study design

All enrolled patients were required to register their complete medical history and undergo demographic analysis, including sex, age, height, weight, drinking history, smoking history, underlying diseases, and long-term medication. The body mass index (BMI) was calculated. The patients received quadruple therapy for *Hp* eradication were prohibited from consuming alcohol during treatment to avoid potential side effects from alcohol interactions with amoxicillin. Avoid foods that irritate the Stomach, such as spicy foods, acidic foods, caffeine, and carbonated drinks. Meanwhile, avoid dairy/calcium-rich foods with Tetracycline—they can interfere with absorption.

Each patient was informed of the common side effects of the drugs before treatment and was instructed to record these symptoms for the evaluation of drug compliance and adverse events. The patients underwent the ^13^C urea breath test 6 weeks after eradication therapy to assess the *Hp* status following treatment.

Fecal samples were collected the day before *Hp* eradication therapy and on day 56, at the end of therapy, for gut microbiota analysis. A small amount of feces (approximately 1 cm^3^) was placed in a 50 mL sterile centrifuge tube containing no preservative fluid. The sample was then sent to the laboratory within 6 h and stored at –80°C for DNA extraction.

The study consisted of two patient visits: one on the day before treatment and another at week 6 after treatment. Each patient was orally informed about the study’s details and they voluntarily signed the informed consent form. The study protocol was reviewed and approved by the hospital’s medical ethics committee (Ethics No. 2021-240).

### 2.2 Inspection method

#### 2.2.1 Microbial DNA extraction and 16SrRNA sequencing of fecal samples

The TIANamp Stool DNA Kit (E.Z.N.A.^®^ Stool DNA Kit, LC-Bio Technologies (Hangzhou) Co., Ltd., Zhejiang) was used. First, the microbiota DNA mass was assessed via agarose gel electrophoresis (AGE), and the DNA concentration was subsequently measured using an ultraviolet spectrophotometer. The study employed V3–V4 regions of the 16S rRNA gene, as these provide optimal resolution for bacterial community profiling in gut microbiota studies.

#### 2.2.2 Bioinformatics analysis

Bioinformatics processing was conducted using a comprehensive pipeline to ensure high-quality data analysis. Initially, sequence quality was assessed using FastQC v0.11.9 to evaluate raw read quality, focusing on per-base Phred scores (Q ≥ 30 required), read length distribution (expected 250 bp paired-end), and guanine-cytosine (GC) content (checked against mock communities). Aggregated reports were generated using MultiQC v1.11 to provide an overview of quality metrics across all samples. Quality control and trimming were performed using the QIIME 2 v2023.2 pipeline with the divisive amplicon denoising algorithm 2 (DADA2) plugin. Adapters were removed using Cutadapt, targeting 5’–3’ Illumina adapters. Reads were trimmed and truncated: Forward reads were trimmed by 10 bp and truncated at 230 bp, while reverse reads were trimmed by 15 bp and truncated at 220 bp. Denoising was conducted with an error rate learning step (maxEE = 2) to correct sequencing errors, followed by chimera removal using the consensus method. For normalization and reference alignment, sequences were aligned against the small subunit rRNA database (SILVA) v138 database [99% operational taxonomic units (OTUs), 515F/806R region] using Multiple Alignment using Fast Fourier Transform (MAFFT) v7.475. Closed-reference OTU picking was performed with a 97% similarity threshold. Sequences were normalized using cumulative sum scaling (CSS) normalization for compositional data and rarefied to 32,000 reads per sample for alpha diversity analysis. Representative sequences were selected using DADA2 denoising to generate exact amplicon sequence variants (ASVs), with length filtering to retain 410–453 bp merged contigs. Verification was conducted using basic local alignment search tool for nucleotide sequences (BLASTn) against the national center for biotechnology information (NCBI) 16S rRNA database to remove non-prokaryotic hits (*e*-value < 1e−50). Taxonomic assignment was performed using BLAST + v2.12.0 against a curated database, with identity thresholds set at ≥ 94% for genus-level (Helicobacter) and ≥ 97% for species-level assignments, and a minimum coverage of ≥ 90% query alignment. The database included a custom combination of SILVA and Helicobacter genomes from NCBI to ensure accurate identification.

#### 2.2.3 Diversity analysis

The Qiime method was used to evaluate the alpha and beta diversity of the fecal microbiota. Alpha diversity reflects the richness (i.e., the number of distinct species) and evenness (i.e., the balance of abundances or the dominance of certain species) within a microbial ecosystem. Beta diversity represents the variations in microbiota composition across different environments. Alpha diversity analyzes the complexity of gut microbiota, commonly using five indexes: Shannon, Simpson, Chao1, Observed species, and Goods coverage. Beta diversity is assessed using the weighted UniFrac distance matrix, and changes in gut microbiota before and after *Hp* eradication therapy are visually analyzed through principal component analysis (PCA). The primary focus of this study was to observe the changes in gut microbiota diversity before and after *Hp* eradication therapy.

#### 2.2.4 Function prediction

Function prediction of the gut microbiota’s 16S rRNA was carried out using PICRUSt2 (Phylogenetic Investigation of Communities by Reconstruction of Unobserved States). The Operational Taxonomic Unit (OTU) abundance table was first normalized, and OTUs were categorized into two groups: The Kyoto Encyclopedia of Genes and Genomes (KEGG) orthologous group and the Cluster of Orthologous Group (COG) orthologous group (Douglas et al., 2020). Using the KEGG database, information on KO, enzymes, and pathways were obtained, and subsequently the abundance of each functional category was calculated based on OTU abundance. Finally, a differential analysis was conducted using the STAMP (Statistical Analysis of Metagenomic Profiles) tool to identify gene functions in microbiota that demonstrated significant differences at various treatment time points.

#### 2.2.5 Statistical analysis

Baseline continuous data were analyzed using one-way analysis of variance (ANOVA), with measurement data expressed as mean ± standard deviation. Categorical data were presented as percentages and compared using the chi-square test or Fisher’s exact test. The Wilcoxon rank sum test was applied to compare the relative abundance of bacterial groups between the patients diagnosed with *Hp*-positive and *Hp*-negative, as well as the diversity and predicted pathway abundance following eradication therapy. For all bacterial groups, the *p*-value threshold was set at 0.05 (Wilcoxon rank sum test), and the effect size threshold was 2. All statistical tests were two-tailed, with *p* < 0.05 indicating statistical significance. In this study, the database was created using Excel, and statistical analysis was performed using SPSS version 20.0.

## 3 Results

### 3.1 General characteristics of patients

A total of 26 patients diagnosed with *Hp*-positive were included in this study, which took place from June 1, 2021, to December 31, 2021. The patients were aged between 19 and 55 years, with a mean age of 36.3 ± 8.84 years. The male-to-female ratio was 9:17. Among these patients, 18 were diagnosed as *Hp*-positive based on positive results from the ^13^C urea breath test, while 8 were diagnosed as *Hp*-positive through gastroscopic histopathologic biopsy. The basic characteristics of the patients are presented in Table 1.

**TABLE 1:** Basic characteristics of patients.

Variable	Total
	26
**Sex**	
Male	9
Female	17
**Age**	
<40 years	16
≥40 years	10
**Height**	
<1.70 m	18
≥1.70 m	8
**Weight**	
< 60 kg	10
≥ 60 kg	16
**BMI (kg/m^2^)**	
< 18.5	1
18.5–23.9	17
≥ 24.0	8
**Smoking history**	
Yes	6
No	20
**Drinking history**	
Yes	11
No	15
**Inspection items**	
^13^C urea breath test	18
Gastroscopic histopathological examination	8

### 3.2 Diversity changes of gut microbiota

#### 3.2.1 Alpha diversity changes

**FIGURE 1:**
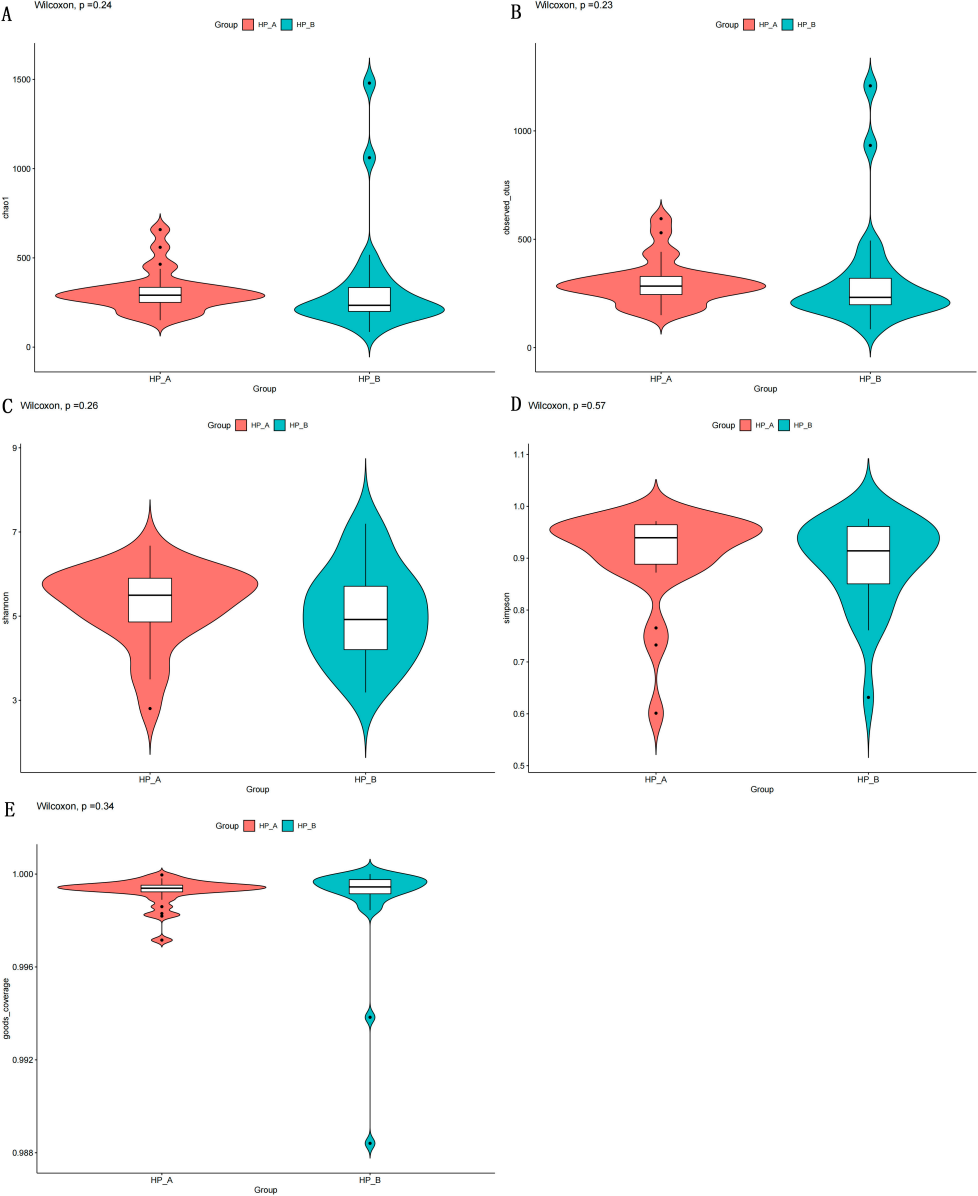
Presents five indices used to evaluate alpha diversity. (A,B) Represent species richness using observed species and Chao1. (C,D) represent species richness and evenness using Shannon and Simpson. (E) Represents sequencing depth using Good’s Coverage. *Hp*-A corresponds to the *Hp*-positive baseline level, while *Hp*-B corresponds to day 56 after *Hp* eradication.

Alpha diversity was analyzed in 2,520 sequences/samples (the minimum sampling depth) to compare microbiota biodiversity at baseline level and 6 weeks after eradication therapy. As presented in Figures 1A,B, no significant differences were observed in the Chao1 index and Observed species index before and after eradication therapy (*p* = 0.24; *p* = 0.23). No significant differences were found in the Simpson index and Shannon index before and after eradication therapy (*p* = 0.57; *p* = 0.26), as presented in Figures 1C,D. No significant difference was found in Good’s Coverage index before and after eradication therapy (*p* = 0.34), as presented in Figure 1E. These results indicated that no significant differences were observed in Alpha diversity changes in fecal microbiota composition 6 weeks after *Hp* eradication therapy (*p* > 0.05), indicating that the Alpha diversity of the gut microbiota did not change significantly 6 weeks after *Hp* eradication therapy.

#### 3.2.2 Beta diversity changes

**FIGURE 2:**
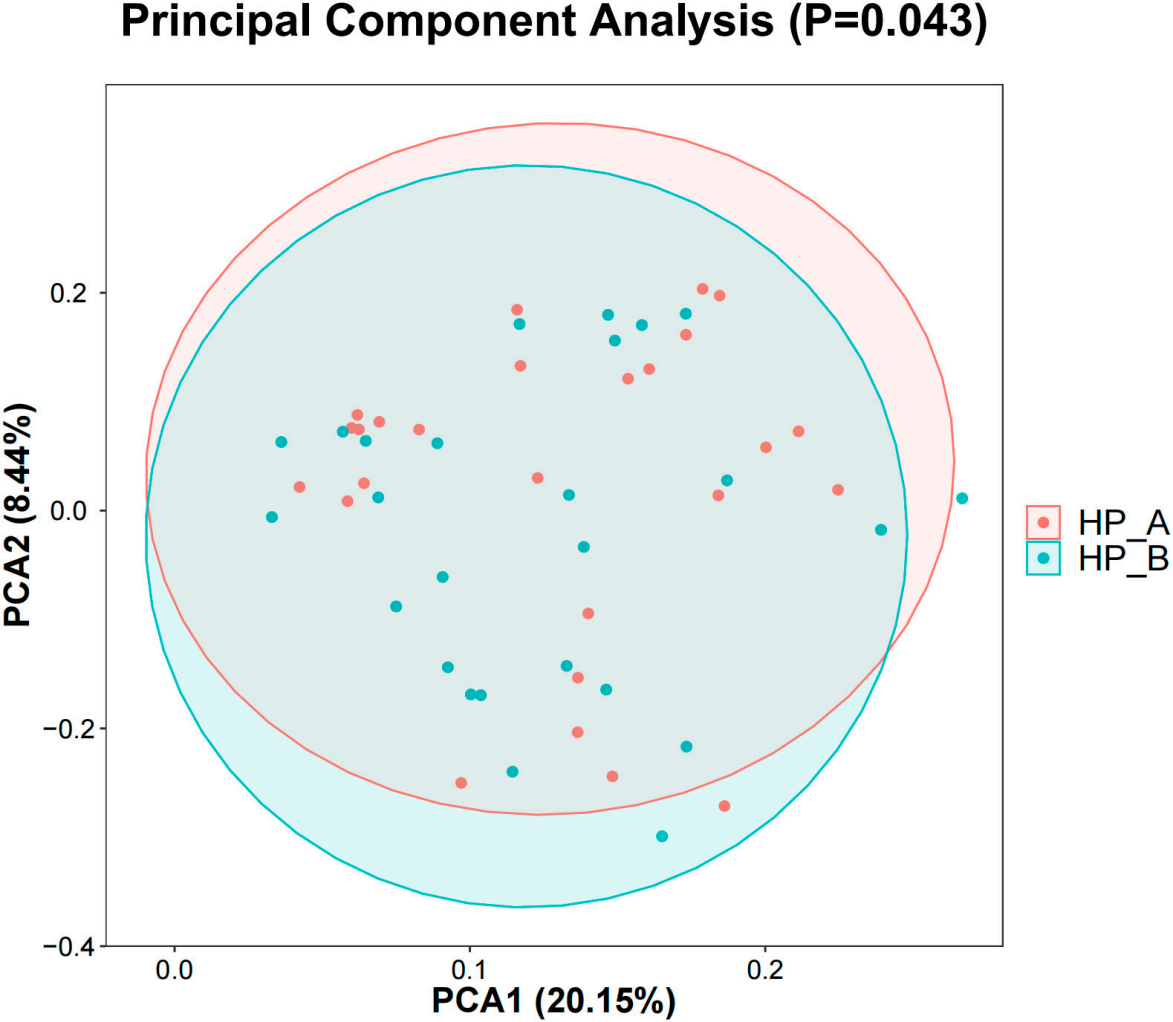
Each point represents a sample, with points that are closer to each other indicating smaller differences in community composition. *Hp*-A is represented by the red point, indicating the *Hp*-positive baseline level, while *Hp*-B is represented by the blue point, indicating the level on day 56 after *Hp* eradication.

The PCA of fecal samples at baseline and 6 weeks after eradication therapy, is presented in Figure 2. By observing the differences in gut microbiota before and after eradication therapy, the samples that were more similar in microbiota composition were positioned closer together (Figure 2). The *p*-value (probability value), calculated from the ANOSIM (analysis of similarity), indicated that significant differences existed in gut microbiota aggregation at baseline and 6 weeks after treatment (*p* = 0.043 < 0.05). This indicates differences in the beta diversity of the gut microbiota on day 56 following eradication therapy.

### 3.3 Analysis on gut microbiota structural composition

**FIGURE 3:**
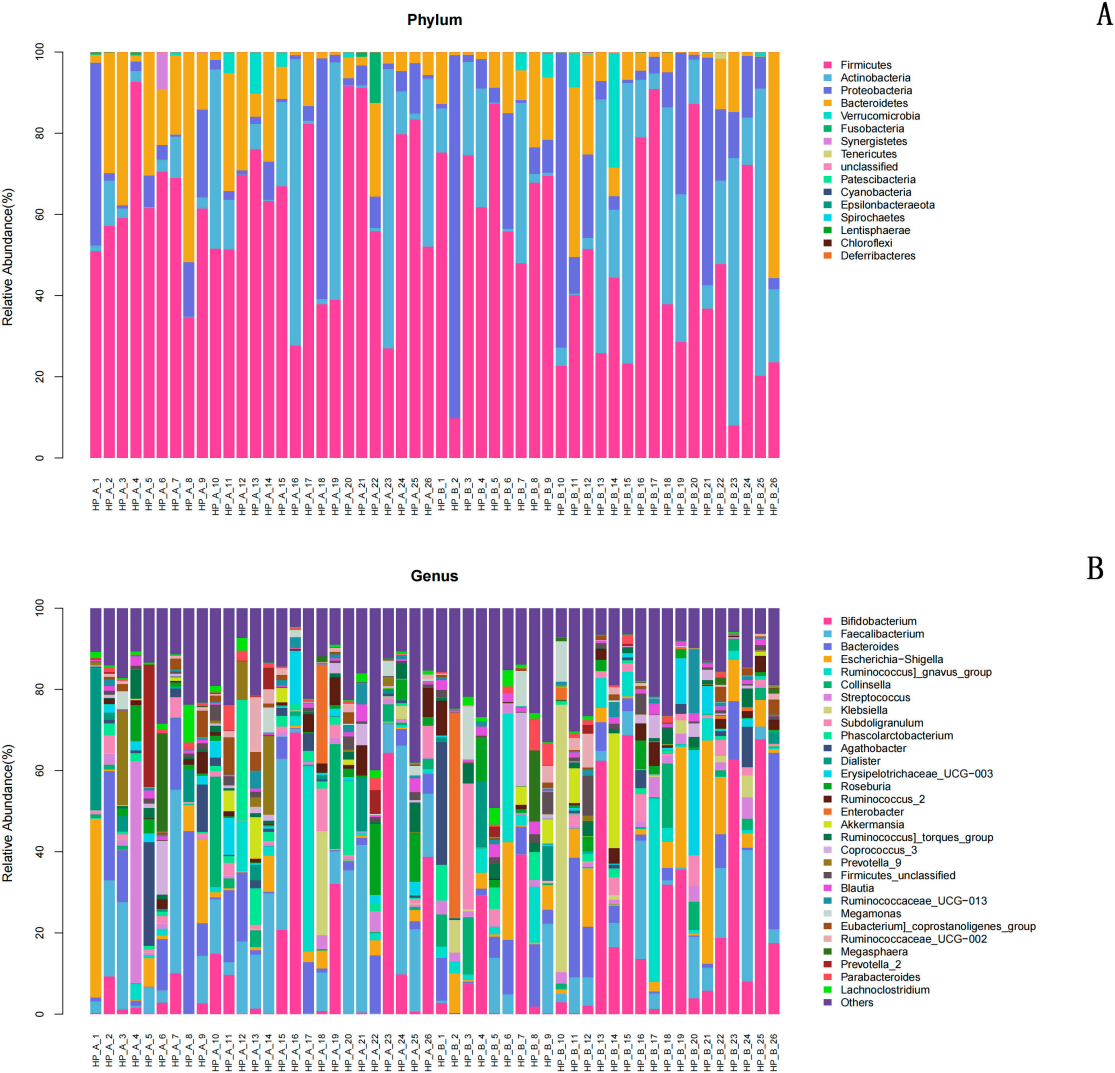
(A) Presents a histogram of relative abundances of phylum-level gut microbiota at the *Hp*-positive baseline level and on day 56 after treatment. On day 56, the relative abundances of Firmicutes and Bacteroidetes decreased, while those of Proteobacteria, Actinobacteria, and Verrucomicrobi*a* increased. *Hp*-A corresponds to the *Hp*-positive baseline level, and *Hp*-B corresponds to day 56 after *Hp* eradication. (B) Presents a histogram of relative abundances of genus-level gut microbiota at the *Hp*-positive baseline level and on day 56 after treatment. At the genus level, the baseline group mainly contained *Faecalibacterium*, *Bifidobacterium*, *Bacteroides*, and *Escherichia-Shigella*, while the group on day 56 included *Bifidobacterium*, *Escherichia-Shigella*, *Bacteroides*, and *Faecalibacterium*. The relative abundance of Bifidobacterium increased. *Hp*-A corresponds to the *Hp*-positive baseline level, and *Hp*-B corresponds to day 56 after *Hp* eradication.

OTUs with 97% similarity were identified from 52 fecal samples. The Wilcoxon rank sum test and the Kruskal-Wallis rank sum test were used to analyze the differences in abundance at all taxonomic levels (phylum, family, class, order, and genus). No *Hp* genus or species were detected in the fecal samples from any of the patients. Significant differences in microbiota abundances were observed among gastrointestinal microbiota groups. At the phylum level, the most abundant phyla in fecal microbiota were Firmicutes, Actinobacteria, Bacteroidetes, and Proteobacteria. Following eradication therapy, the relative abundances of Firmicutes and Bacteroidetes decreased, while those of Proteobacteria, Actinobacteria, and Verrucomicrobia increased. The results are presented in Figure 3.

#### 3.3.1 Changes in relative abundance of each phylum

**FIGURE 4:**
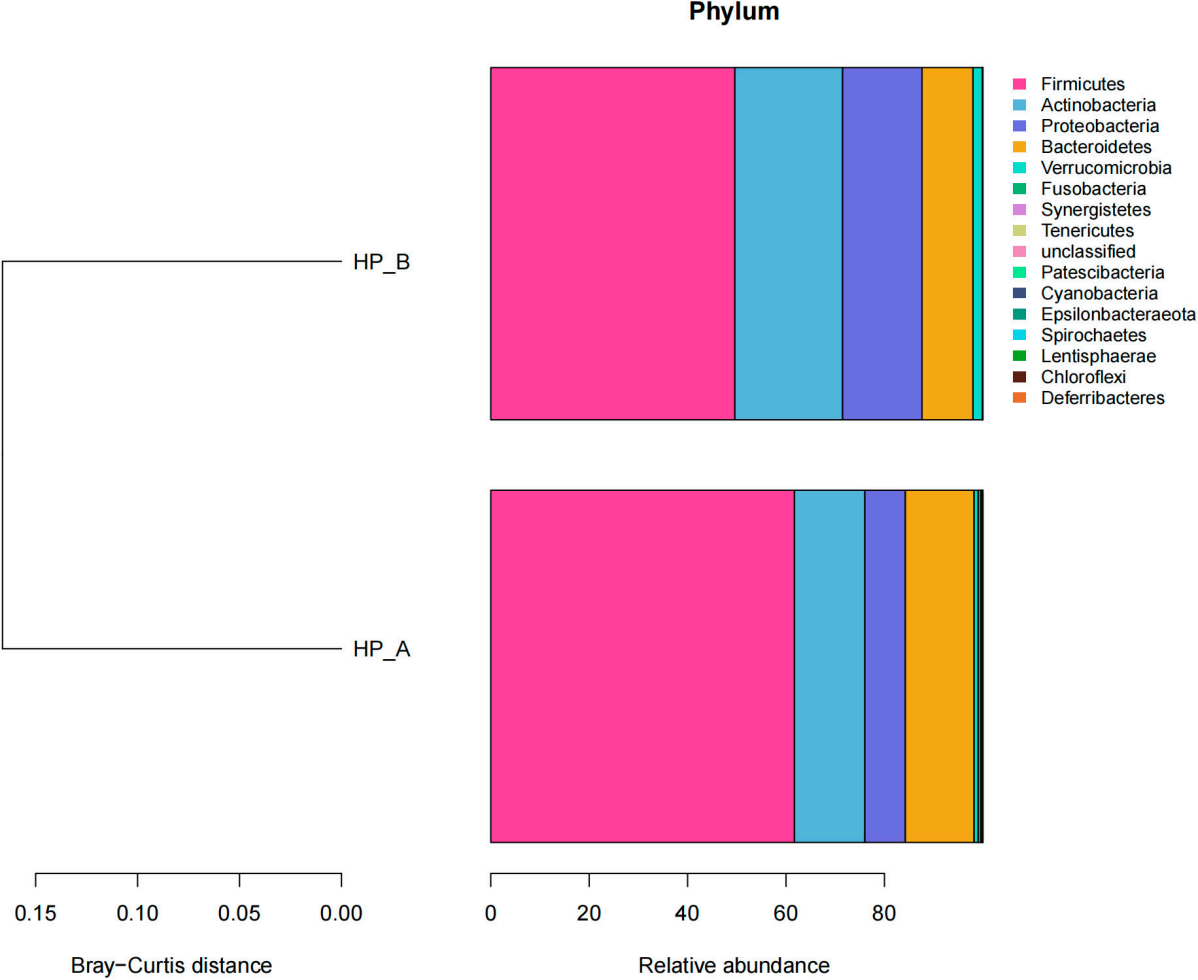
presents a histogram of relative abundances of phylum-level gut microbiota at the baseline level and on day 56 after treatment. *Hp*-A corresponds to the *Hp*-positive baseline level, and *Hp*-B corresponds to day 56 after *Hp* eradication.

Significant changes were observed in the gut microbiota at the phylum-level following Helicobacter pylori (Hp) eradication therapy (Figure 4).

Demonstrates the relative abundances of phylum-level gut microbiota in the baseline group and on day 56 after eradication. At the phylum level, we observed nominal changes in the relative abundances of key taxa following Helicobacter pylori (*Hp*) eradication therapy, although most differences were not statistically significant after accounting for biological variability. Specific changes are as follows:

- The relative abundance of Firmicutes decreased from 61.7% (± 5.2) to 49.6% (± 6.1) (ANCOM-II, *q* = 0.08);
- The relative abundance of Actinobacteria increased from 14.3% (± 3.8) to 21.9% (± 4.5) (ANCOM-II, *q* = 0.06);
- The relative abundance of Proteobacteria increased significantly from 8.2% (± 2.9) to 16.2% (± 4.1) (ANCOM-II, *q* = 0.04, statistically significant);
- The relative abundance of Bacteroidetes decreased from 14.0% (± 3.6) to 10.4% (± 3.2) (ANCOM-II, *q* = 0.12).

The Bacteroidetes/Firmicutes (B/F) ratio increased from 4.42 (± 1.8) to 4.79 (± 2.1) (ANCOM-II, *q* = 0.41), with no statistical significance. Further paired analysis showed the pre-treatment B/F ratio was 4.4 (95% CI: 3.1–5.9), and the post-treatment ratio was 4.8 (95% CI: 3.3–6.5). The paired difference was + 0.4 (95% CI: –1.2 to + 2.0, *p* = 0.61; ANCOM-II, *q* = 0.72), confirming no statistically significant change in the B/F ratio after Hp eradication. The apparent 9% increase fell well within the expected biological variability of this metric, as supported by overlapping confidence intervals.

Minor phyla also exhibited changes:

- The relative abundance of Verrucomicrobia increased from 0.83% (± 0.7) to 1.88% (± 1.1) (ANCOM-II, *q* = 0.23);
- The relative abundance of Fusobacteria decreased from 0.58% (± 0.4) to 0.02% (± 0.02) (ANCOM-II, *q* = 0.15).

These results suggest that while there were notable shifts in the relative abundances of several phyla following *Hp* eradication, only the increase in *Proteobacteria* reached statistical significance (*q* = 0.04).

#### 3.3.2 Changes in relative abundance of each genus

**FIGURE 5:**
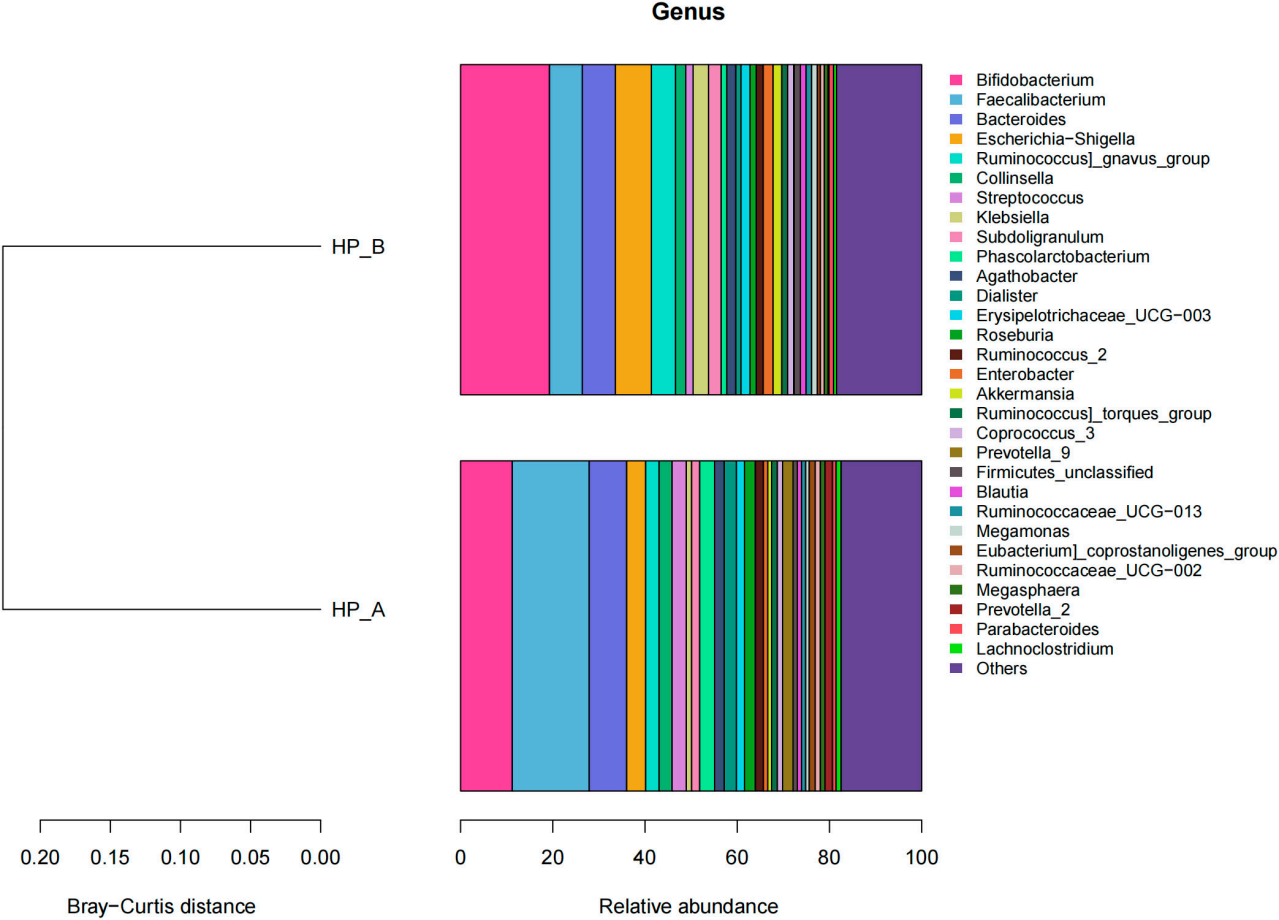
presents a histogram of relative abundances of genus-level gut microbiota at the baseline level and on day 56 after treatment. *Hp*-A corresponds to the *Hp*-positive baseline level, and *Hp*-B corresponds to day 56 after *Hp* eradication.

The relative abundances of genus-level gut microbiota in the baseline group and on day 56 after eradication therapy are presented in Figure 5. At the genus level, the fecal microbiota primarily consisted of *Faecalibacterium* (16.70%), *Bifidobacterium* (11.20%), *Bacteroides* (8.12%), and *Escherichia-Shigella* (4.12%) in the *Hp* baseline group, and *Bifidobacterium* (19.29%), *Escherichia-Shigella* (7.81%), *Bacteroides* (7.18%), *Ruminococcus* (5.23%), and *Faecalibacterium* (7.11%) on day 56 after treatment. At the genus level, the fecal microbiota primarily consisted of *Faecalibacterium, Bifidobacterium, Bacteroides, and Escherichia-Shigella* in the *Hp* baseline group, and *Bifidobacterium, Escherichia-Shigella, Bacteroides, Ruminococcus, and Faecalibacterium* on day 56 after treatment.

The relative abundance of *Bacteroides* in *Bacteroidetes* decreased from 8.12 to 7.11%, *Streptococcus* from 3.08 to 1.59%, *Bacillus* from 3.23 to 1.22%, and *Faecalibacterium* in *Firmicutes* from 16.70 to 7.11%. In *Firmicutes*, the relative abundance of *Ruminococcus* increased from 2.92 to 5.23%, *Escherichia-Shigella* from 4.12 to 7.81%, and *Klebsiella* from 1.16 to 3.36%. At the genus level, the relative abundance of the commensal *Bifidobacterium* significantly increased, as did that of *Escherichia-Shigella, Ruminococcus*, and *Klebsiella*, while *Faecalibacterium, Bacteroides, Bacillus, Streptococcus, Haemophilus*, and *Prevotella* decreased on day 56 after treatment.

### 3.4 Analysis on microbiota difference before and after *Hp* eradication

**FIGURE 6:**
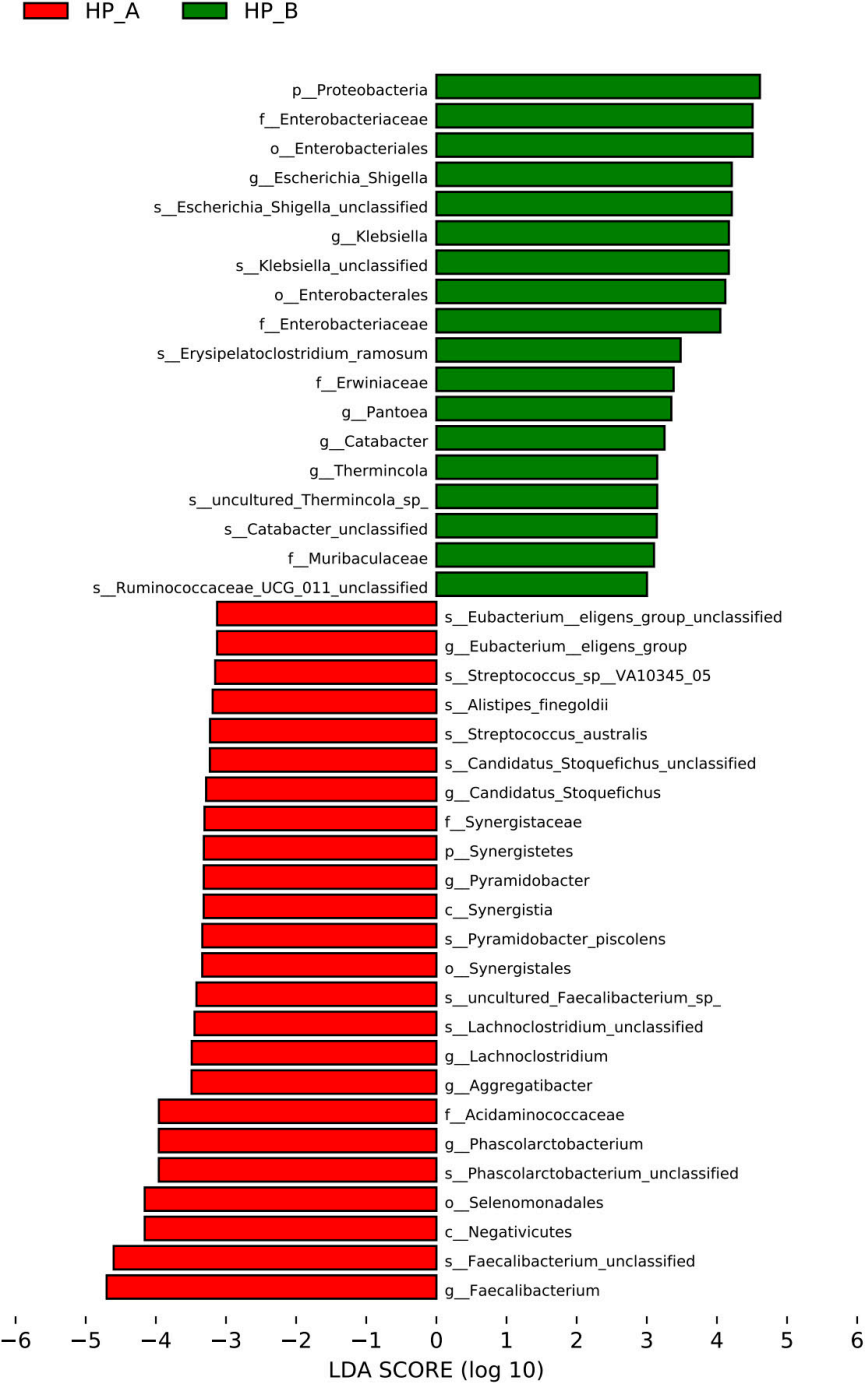
presents the histogram of LDA value distribution before and after treatment, with the bar length representing the LDA value. The chart highlights species with significant differences (LDA value > 3), indicating biomarkers with significant differences. The red bar in the histogram represents *Hp*-A, corresponding to the baseline level, and the green bar represents *Hp*-B, corresponding to day 56 after *Hp* eradication. The longer the bar, the greater the impact of species significantly different before and after treatment.

According to LEfSe (Figure 6), significant differences in the abundances of microbiota were observed from the phylum to the species level. Figure 6 displays the linear discriminant analysis (LDA) score histogram for microbiota with significant differences at the phylum and genus levels before and after treatment. It was revealed in the LDA score that, at the phylum level, the gut microbiota predominantly consisted of Firmicutes, Actinobacteria, and Bacteroidetes at the *Hp*-positive baseline, while Firmicutes, Proteobacteria, and Actinobacteria predominated on day 56 after eradication therapy. At the genus level, *Faecalibacterium, Bifidobacterium, Bacteroides*, and *Escherichia-Shigella* were most abundant at the *Hp*-positive baseline, whereas *Bifidobacterium, Escherichia-Shigella, Bacteroides, Ruminococcus*, and *Faecalibacterium* were predominant on day 56 after treatment. These results indicate significant differences in the gut microbiota composition between the groups before and after *Hp* eradication.

At the phylum level (L1), significant changes were observed in the gut microbiota composition following Helicobacter pylori (Hp) eradication therapy. Specifically, *Firmicutes* and *Actinobacteria* were identified as true positives, with *q*-values of 0.008 and 0.01, respectively, indicating that these changes passed the False Discovery Rate (FDR) correction threshold. In contrast, *Bacteroidetes* did not meet the significance threshold (*q* = 0.07) and was therefore removed from the list of significant taxa.

At the genus level (L6), several key genera exhibited significant changes in relative abundance between the baseline and post-treatment (Day 56) time points. *Faecalibacterium* showed a substantial decrease from 16.7 to 7.1%, with an LDA score of 4.1 and a q-value of 0.003. *Escherichia* increased from 4.1 to 7.8%, with an LDA score of 3.8 and a q-value of 0.02. *Bifidobacterium* also increased significantly, from 11.2 to 19.3%, with an LDA score of 3.5 and a *q*-value of 0.04. These findings highlight the dynamic shifts in microbial community structure following Hp eradication therapy, with notable changes observed at both the phylum and genus levels.

### 3.5 Prediction of changes in microbiota function

**FIGURE 7:**
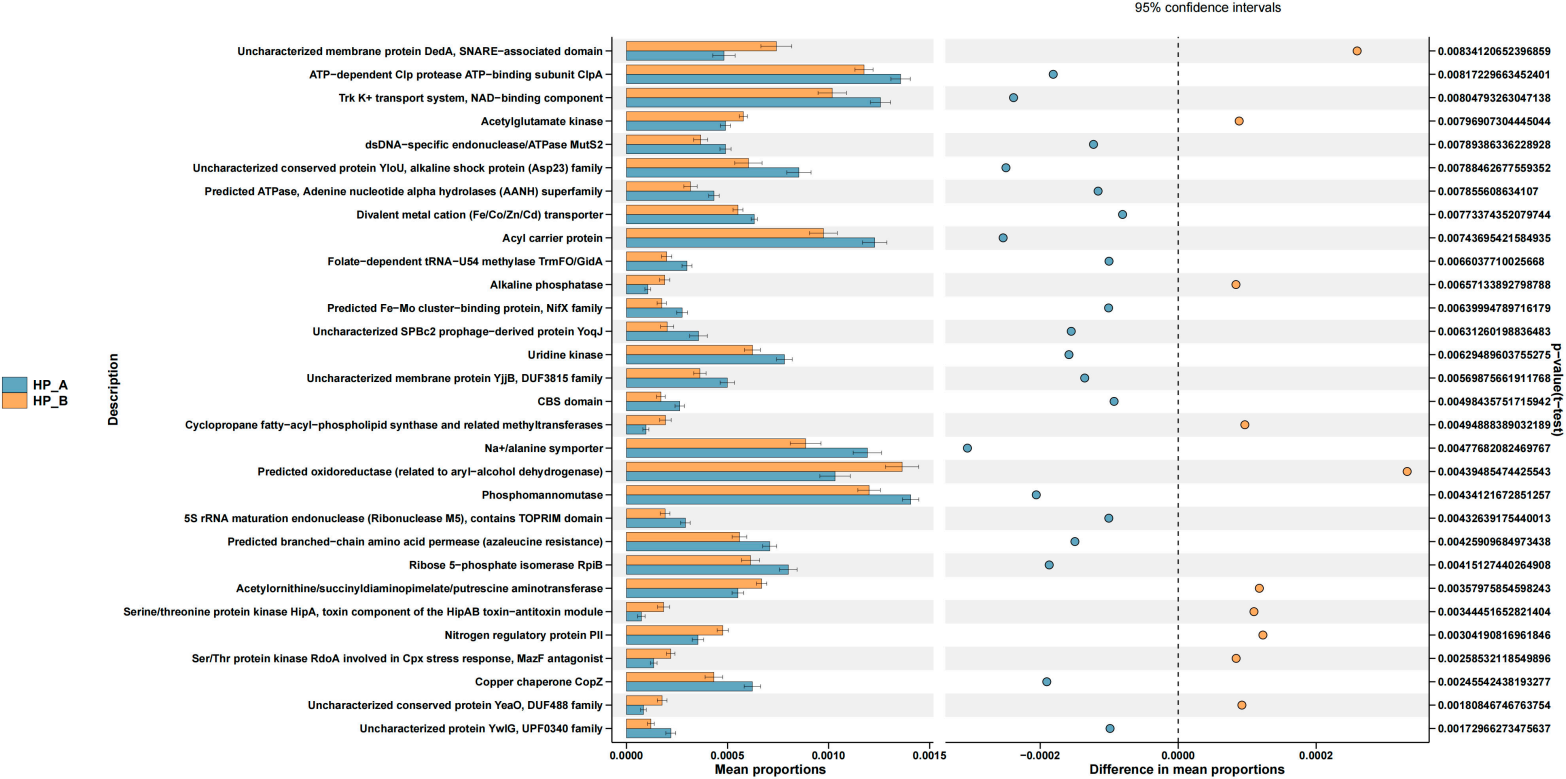
compares the functional annotation results from the COG database before and after treatment, selecting functions with significant differences between groups. The blue section represents *Hp*-A, while the orange section represents *Hp*-B. The horizontal bar on the left indicates the abundance of enrichment in this metabolic pathway, representing the percentage of all metabolic pathways at the two treatment time points, with the corrected *p-*value shown on the right. *Hp*-A, at the *Hp*-positive baseline level; *Hp*-B, on day 56 after *Hp* eradication.

The functional potential of the gut microbiota was inferred using PICRUSt2, a tool that predicts metagenomic functional content from 16S rRNA gene sequences and a reference genome database. The predicted metagenomes were analyzed against the KEGG database. As presented in Figure 7, significant differences were observed in the relative abundances of 30 pathways between the baseline level and day 56 after eradication therapy, according to KEGG pathway analysis. In the human diseases class, the abundance of cancer-related pathways was higher at the *Hp*-positive baseline, while the abundance of metabolic disease pathways decreased following *Hp* eradication therapy. In the metabolism class, the abundances of amino acid, lipid, and carbohydrate metabolic pathways were lower on day 56 after *Hp* eradication compared to the baseline group.

## 4 Analysis and discussion

### 4.1 Basic conditions of *Hp*-colonized gut microbiota

The human gut microbiota consists of more than 10–100 trillion symbiotic microbes, which play a critical role in the body’s metabolic, physiological, and immune systems (Heimesaat et al., 2014). Studies have demonstrated that *Hp* infection may alter the gastrointestinal microbiota by disrupting the immune, mechanical, and biological barriers, as well as other functions of the gastrointestinal mucosa (Cover and Blaser, 2009; Frost et al., 2019). Cytotoxin-associated gene A, a virulence factor secreted by *Hp*, can disturb the ecological balance of the gut microbiota. Furthermore, *Hp* may induce the secretion of inflammatory cytokines or neuroendocrine mediators, which can regulate the gut microbiota at non-inflammatory sites, subsequently triggering host immune responses.

In this study, the most abundant phyla in the fecal samples from patients with *Hp*-infection included Firmicutes, Actinobacteria, Bacteroidetes, and Proteobacteria, while the relative abundance of *Klebsiella, Coprococcus, Ruminococcus, and Staphylococcus* was lower. At the genus level, the fecal microbiota primarily consisted of *Faecalibacterium, Bifidobacterium, Bacteroides*, and *Escherichia-Shigella* in the *Hp*-positive baseline group. Other studies have indicated that the gut microbiota of patients with *Hp*-infection is rich in members of *Succinivibrionaceae, Coriobacteriaceae, Enterococcaceae*, and the *Pyrenomycetes* family (Treiber et al., 2003). In patients with *Hp*-infection, the abundance of Proteobacteria significantly increased, while the relative abundance of Firmicutes and Bacteroidetes remained high, resulting in an elevated *B/F* ratio. Research has indicated that changes in the *B/F* ratio may be involved in metabolic diseases, with a significant decrease in the level of short-chain fatty acids produced in infected adults. There is a noted correlation between *Hp* infection and gut microbiota diversity. The observed Proteobacteria increase aligns with *H. pylori*’s disruption of gastric acid barriers (Chmiela and Kupcinskas, 2019), permitting enteric pathogen colonization. Notably, the absence of detectable fecal *H. pylori* (despite gastric infection) mirrors Kumar et al. (2021), suggesting minimal intestinal translocation in our cohort. Our observed B/F ratio increase (4.42→4.79) may reflect *H. pylori*-induced metabolic disturbances. A pivotal finding was the profound decline in Faecalibacterium prausnitzii, a primary butyrate producer. Butyrate is crucial for colonocyte energy metabolism, maintenance of the gut barrier integrity, and possesses anti-inflammatory properties (Miquel et al., 2013). Its depletion suggests a compromised colonic environment post-therapy, potentially predisposing patients to inflammation and impaired gut barrier function. This may mechanistically explain the abdominal discomfort or transient dysbiosis symptoms reported by some individuals following eradication therapy (Młynarska et al., 2024), and aligns with the understanding of *F. prausnitzii* as a keystone taxon for gut health (Lopez-Siles et al., 2017).

Conversely, the expansion of Escherichia-Shigella, a genus encompassing opportunistic pathogens, highlights a shift in the microbial ecology. This bloom is likely facilitated by the ecological vacancy created by the broad-spectrum antibiotics, which suppress competing commensals. The increase in such proteobacterial pathobionts is clinically significant, as it may not only contribute to post-therapy symptoms but also represent a state of elevated risk for opportunistic infections or sustained low-grade inflammation, underscoring the need for monitoring and potential restorative interventions.

### 4.2 Effect of *Hp* eradication on gut microbiota composition

The gut microbiota composition underwent significant changes following *Hp* eradication, as demonstrated in this study. At the phylum level, the relative abundance of Firmicutes, Bacteroidetes, and Verrucomicrobia (Fusobacteria) decreased, while the relative abundance of *Proteobacteria* increased after eradication therapy. At the genus level, the fecal microbiota primarily consisted of *Faecalibacterium, Bifidobacterium, Bacteroides, and Escherichia-Shigella* in the *Hp*-positive baseline group, and *Bifidobacterium, Escherichia-Shigella, Bacteroides*, and *Faecalibacterium* on day 56 post-therapy (Figure 3B). Notably, the commensal *Bifidobacterium* demonstrated an increase in relative abundance. Proteobacteria increased from 8.2 to 16.2% (*p* = 0.01), while Firmicutes decreased from 61.7 to 49.6% (*p* = 0.008). Bifidobacterium* abundance rose from 11.2 to 19.3% (*p* = 0.003), whereas Faecalibacteriumdeclined from 16.7 to 7.1% (*p* = 0.001). Our observed Enterobacteriaceae increase aligns with Liu et al. (2022), who reported significant Proteobacteria expansion and reduced microbial diversity following first-line *H. pylori* eradication therapy. Additionally, the abundance of gut symbionts and microbiota diversity significantly decreased. First-line *Hp* eradication therapy typically includes two antibiotics and bismuth. Some patients may experience symptoms such as diarrhea, constipation, abdominal distension, and even dysbiosis following treatment (Uemura et al., 2001). Additionally, excessive exposure to antibiotics can lead to resistance in the resident microbiota, which may then transfer to pathogenic bacteria (Dong and Liang, 2015; Ishaq and Nunn, 2015). Tetracycline and amoxicillin are broad-spectrum antibiotics that can directly damage gastrointestinal epithelial cells and promote the spread of drug-resistant microbes, thereby affecting other harmful bacteria during *Hp* eradication (Dhariwal et al., 2022; Gokulan et al., 2017; Jorth et al., 2021). Moreover, significant changes were observed in gut microbiota diversity and composition after *Hp* eradication. These patterns align with but refine Hsu et al.’s observations, showing dysbiosis is common yet modifiable rather than inevitable. Clinical monitoring should focus on high-risk subgroups.

In this study, only the changes in gut microbiota on day 56 after *Hp* eradication therapy were observed. The results indicated that the relative abundances of Firmicutes, Bacteroidetes, and Fusobacteria decreased compared to baseline levels, which aligns with the findings of Hsu et al. (2018). The time point of “week 6 after eradication therapy” was adopted from previous studies and serves as the routine follow-up time for patients undergoing eradication therapy (Zhou et al., 2022; Ishizuka et al., 1999; Malfertheiner et al., 2022; Chen et al., 2024). This time point is advantageous for sample collection and regular monitoring of patients.

The rise in B/F ratio (4.42→4.79) aligns with established links to reduced short-chain fatty acids (SCFA) production and metabolic dysregulation (Morrison and Preston, 2016). This mirrors findings in antibiotic-treated cohorts without *H. pylori*, suggesting therapy-driven effects. While *H. pylori* (Phylum: Campylobacterota) was undetectable in fecal metagenomes, its gastric niche may indirectly shape gut microbiota via: Systemic inflammation elevating Proteobacteria (Buzás, 2018). Antibiotic exposure selecting for resistant Enterobacteriaceae (Figure 4). These mechanisms are distinct from direct phylum-level competition.

### 4.3 Effect of *Hp* eradication on gut microbiota diversity

In this study, differences in the beta diversity of gut microbiota following *Hp* eradication were observed, while no significant differences were observed in alpha diversity. After eradication therapy, the microbiota diversity altered by *Hp* infection appeared to be restored. In the short follow-up period, the relative abundance of Firmicutes, Bacteroidetes, and Fusobacteria decreased, while the abundance of Actinobacteria, Verrucomicrobia, and Proteobacteria increased. At the genus level, the relative abundance of *Bifidobacterium, Escherichia-Shigella, Ruminococcus*, and *Klebsiella* increased, while *Faecalibacterium, Bacteroides, Bacillus, and Streptococcus* decreased.

In this study, changes in alpha diversity of the gut microbiota were not significant on day 56 after eradication, suggests a complex ecological response, which could be attributed to two possible scenarios. First, he stability of alpha diversity at Day 56 (p* = 0.12) may reflect: rapid recovery (undetected without interim timepoints), *or* baseline resilience in our cohort (high *Bifidobacterium).* Second, alpha diversity may have remained unchanged after eradication. These findings could be influenced by the small sample size, and both the sample size and follow-up duration will need to be expanded in future studies. A literature review indicated that no further changes in gut microbiota were observed after more than 6 months, and the microbiota composition returned to baseline levels 2 years post-eradication (Zhao et al., 2019). Additionally, no significant differences were found in the relative abundance of genus-level microbiota before and after eradication (Liou et al., 2019).

The impact of *Hp* eradication on the microbiota should be considered when treating *Hp* infection. Due to ethical constraints, the relatively small cohort size (*n* = 26) may limit the statistical power to detect more subtle shifts in microbial composition and reduces the generalizability of our findings to broader populations. Future studies with larger, multi-center cohorts are warranted to validate these preliminary findings and to explore the heterogeneity of gut microbiota responses to *H. pylori* eradication therapy.

### 4.4 Prediction of microbiota function

The functional changes in gut microbiota at the *Hp*-positive baseline level and at week 6 after *Hp* eradication was predicted in this study, based on microbiota abundance and the KEGG database. The results indicated a high abundance of infectious disease and cancer pathways at the *Hp*-positive baseline. Infectious disease pathways were upregulated, while cancer pathways were consistent with the clinical pathogenicity of *Hp*, which is associated with both *Hp* infection and gastric cancer. On day 56 after eradication, the abundance of amino acid, lipid, and carbohydrate metabolic pathways was reduced. The reduction in carbohydrate and lipid metabolic pathways was linked to the significant decrease in Firmicutes and Bacteroidetes, key players in dietary fiber fermentation. This suggests a reduced capacity for microbial synthesis of short-chain fatty acids (SCFAs), which is consistent with the observed depletion of Faecalibacterium. SCFAs are not only a primary energy source for the colon but also regulate systemic metabolism and immune function. Therefore, this functional decline could have implications for long-term metabolic health in frequently treated patients, warranting further investigation. The decrease in both Firmicutes and Bacteroidetes after *Hp* eradication may also predict a decline in lipid metabolism. Additionally, the increase in the *B/F* ratio after *Hp* eradication may influence metabolism. Moreover, the eradication regimen, which includes two antibiotics, disrupt gut microbiota homeostasis, leading to short-term alterations in the healthy microbiota and potentially causing long-term changes in its composition and function. Interestingly, a higher abundance of the colorectal cancer pathway in the *Hp* baseline group was indicated in this study. In line with existing literature (Epplein et al., 2013; Kountouras et al., 2018; Kumar et al., 2018; Myllyluoma et al., 2007; Oh et al., 2016), our cohort showed numerical trends consistent with known *H. pylori*-colorectal associations.

The most important limitation of this study is the lack of a control group. While our longitudinal design robustly captures changes from baseline within the same individuals, we cannot definitively partition the observed effects into those specifically caused by the elimination of *H. pylori* versus those resulting from the direct impact of the antibiotics on the commensal microbiota. This is a common challenge in clinical studies of infectious disease eradication. Therefore, our findings should be interpreted as describing the net change in the gut ecosystem following the therapeutic intervention as a whole.

It is crucial to note that our functional analysis using PICRUSt2 provides predictions of metabolic potential rather than direct measurements of functional activity. While this approach is valuable for generating hypotheses from 16S rRNA data, its inferences are limited by the completeness of the reference database and may not fully capture the *in situ* functional capacity of the microbial community. The observed shifts in predicted pathways, such as the reduction in carbohydrate and lipid metabolism, should therefore be interpreted as pointing toward potential functional changes that warrant validation in future studies through direct methods like shotgun metagenomic sequencing or metabolomic profiling.

## 5 Conclusion

This prospective study aimed to evaluate the impact of Helicobacter pylori (*Hp)* eradication on gut microbiota composition. We compared the gut microbiota profiles of participants before and after bismuth-based quadruple therapy, with follow-up assessments conducted on day 56 post-eradication. According to the findings, the abundance of the gut microbiota decreased, and its diversity changed on day 56 following eradication therapy. On day 56 after treatment, beta diversity changed significantly, while alpha diversity demonstrated no significant changes compared to the baseline level. At both the phylum and genus levels, notable shifts in microbial relative abundances were observed, with the most prominent being a statistically significant increase in Proteobacteria (ANCOM-II, *q* = 0.04) among phyla, and a significant elevation in the commensal genus Bifidobacterium among genera.

This study comprehensively characterizes longitudinal changes in gut microbiota composition and predicted function following *H. pylori (Hp)* eradication with bismuth-based quadruple therapy. However, group-level “before vs. after” comparisons may obscure inter-individual response heterogeneity. We thus propose a *post hoc* analysis to categorize patients into subpopulations (e.g., “Severe Dysbiosis,” “Moderate Change,” “Resilient”) based on microbiota alteration magnitude, to uncover distinct response patterns and enhance clinical relevance.

Notably, the study lacks age- and sex-matched control groups stratified by *Hp* infection (infected vs. non-infected) and treatment exposure (treated vs. non-treated), limiting the isolation of *Hp* eradication-specific effects from confounders. To address this and advance personalized medicine, we suggest shifting from epidemiological to individual-level analyses: tracking longitudinal microbiota changes per patient, quantifying individual-level microbial co-occurrence patterns (positive/negative), and stratifying by sex and age to identify demographic-specific trajectories.

Future studies with controlled designs—including comparisons of different *Hp* eradication regimens, or the inclusion of *Hp*-negative control groups receiving similar antibiotics for other indications—are warranted to disentangle the specific contribution of *Hp* elimination from the confounding effects of antibiotics on gut microbiota alterations.

## Data Availability

The original contributions presented in the study are publicly available. This data can be found here: National Center for Biotechnology Information (NCBI) under the BioProject number PRJNA1365776.

## References

[B1] Buzás G. M. (2018). *Helicobacter* pylori in human health and disease: Mechanisms for local gastric and systemic effects. *Cell. Mol. Life Sci.* 75 2985–3009. 10.3748/wjg.v24.i28.3071 PMC6064966 30065554

[B2] Chen H. Duan X. Zhang Y. Chen S. Lv J. Liang X. (2024). 14-day tailored PCR-guided triple therapy versus 14-day non-bismuth concomitant quadruple therapy for Helicobacter pylori eradication: A multicenter, open-label randomized noninferiority controlled trial. *Helicobacter* 29:e13093. 10.1111/hel.13093 38680067

[B3] Chen J. Li P. Huang Y. Guo Y. Ding Z. Lu H. (2022). Primary antibiotic resistance of *Helicobacter* pylori in different regions of China: A systematic review and meta-analysis. *Pathogens* 11:786. 10.3390/pathogens11070786 35890031 PMC9316315

[B4] Chmiela M. Kupcinskas J. (2019). Review: Pathogenesis of *Helicobacter* pylori infection. *Helicobacter* 24:e12638. 10.1111/hel.12638 31486234 PMC6771490

[B5] Coelho L. Coelho M. (2021). *Helicobacter* pylori and colorectal neoplasms: A concise review. *Arq. Gastroenterol.* 58 114–119. 10.1590/S0004-2803.202100000-19 33909789

[B6] Cover T. Blaser M. (2009). *Helicobacter* pylori in health and disease. *Gastroenterology* 136 1863–1873. 10.1053/j.gastro.2009.01.073 19457415 PMC3644425

[B7] Dhariwal A. Bråten L. C. H. Sturød K. Ravi A. Hjerde E. Afset J. E. (2022). Differential response to prolonged amoxicillin treatment: Long-term resilience of the microbiome versus long-lasting perturbations in the gut resistome. *Gut Microbes* 15:2157200. 10.1080/19490976.2022.2157200 36576106 PMC9809947

[B8] Dong H. X. Liang H. (2015). Correlation between colorectal cancer and *Helicobacter* pylori infection in different countries: A meta-analysis. *Med. J. Chinese People’s Liberation Army* 40 236–241. 10.11855/j.issn.0577-7402.2015.03.13

[B9] Douglas G. Maffei V. Zaneveld J. Yurgel S. Brown J. Taylor C. (2020). PICRUSt2 for prediction of metagenome functions. *Nat. Biotechnol.* 38 685–688. 10.1038/s41587-020-0548-6 32483366 PMC7365738

[B10] Epplein M. Pawlita M. Michel A. Peek R. Cai Q. Blot W. (2013). *Helicobacter* pylori protein-specific antibodies and risk of colorectal cancer. *Cancer Epidemiol. Biomarkers Prev.* 22 1964–1974. 10.1158/1055-9965.EPI-13-0702 24045925 PMC3828745

[B11] Fishbein S. R. S. Mahmud B. Dantas G. (2023). Antibiotic perturbations to the gut microbiome. *Nat. Rev. Microbiol.* 21 772–788. 10.1038/s41579-023-00933-y 37491458 PMC12087466

[B12] Frost F. Kacprowski T. Rühlemann M. Bang C. Franke A. Zimmermann K. (2019). *Helicobacter* pylori infection associates with fecal microbiota composition and diversity. *Sci. Rep.* 9:20100. 10.1038/s41598-019-56631-4 31882864 PMC6934578

[B13] Gokulan K. Cerniglia C. Thomas C. Pineiro S. Khare S. (2017). Effects of residual levels of tetracycline on the barrier functions of human intestinal epithelial cells. *Food Chem. Toxicol.* 109 253–263. 10.1016/j.fct.2017.09.004 28882639

[B14] Heimesaat M. Fischer A. Plickert R. Wiedemann T. Loddenkemper C. Göbel U. (2014). *Helicobacter* pylori induced gastric immunopathology is associated with distinct microbiota changes in the large intestines of long-term infected Mongolian gerbils. *PLoS One* 9:e100362. 10.1371/journal.pone.0100362 24941045 PMC4062524

[B15] Hooi J. Lai W. Ng W. Suen M. Underwood F. Tanyingoh D. (2017). Global prevalence of *Helicobacter* pylori infection: Systematic review and meta-analysis. *Gastroenterology* 153 420–429. 10.1053/j.gastro.2017.04.022 28456631

[B16] Hsu P. Pan C. Kao J. Tsay F. Peng N. Kao S. (2018). *Helicobacter* pylori eradication with bismuth quadruple therapy leads to dysbiosis of gut microbiota with an increased relative abundance of *Proteobacteria* and decreased relative abundances of Bacteroidetes and Actinobacteria. *Helicobacter* 23:e12498. 10.1111/hel.12498 29897654

[B17] IARC Working Group on the Evaluation of Carcinogenic Risks to Humans. (1994). Schistosomes, liver flukes and *Helicobacter* pylori. *IARC Monogr. Eval. Carcinogenic Risks Humans* 61 1–241. PMC7681621 7715068

[B18] Ishaq S. Nunn L. (2015). *Helicobacter* pylori and gastric cancer: A state of the art review. *Gastroenterol. Hepatol. Bed Bench.* 8 S6–S14. 10.22037/ghfbb.v8iSupplement.653 26171139 PMC4495426

[B19] Ishizuka J. Kato M. Sugiyama T. Asaka M. (1999). The appropriate time for the assessment of *Helicobacter* pylori eradication. *Nihon Rinsho.* 57 111–115. 10036946

[B20] Jorth P. Q. Srinivasan R. Merenbloom J. C. Kalra A. Patel S. Whiteley M. (2021). Species interactions drive the spread of ampicillin resistance in human-associated gut microbiota. *mBio* 12:e01649–21. 10.1128/mBio.01649-21 PMC8385247 34447576

[B21] Kamada N. Seo S. Chen G. Núñez G. (2013). Role of the gut microbiota in immunity and inflammatory disease. *Nat. Rev. Immunol.* 13 321–335. 10.1038/nri3430 23618829

[B22] Kountouras J. Polyzos S. Doulberis M. Zeglinas C. Artemaki F. Vardaka E. (2018). Potential impact of *Helicobacter* pylori-related metabolic syndrome on upper and lower gastrointestinal tract oncogenesis. *Metabolism* 87 18–24. 10.1016/j.metabol.2018.06.008 29936174

[B23] Kumar A. Kim M. Lukin D. (2018). *Helicobacter* pylori is associated with increased risk of serrated colonic polyps: Analysis of serrated polyp risk factors. *Indian J. Gastroenterol.* 37 235–242. 10.1007/s12664-018-0855-8 29876742

[B100] Kumar N. Mohan A. Bajpai P. Panda A. Gupta V. Ahuja V. (2021). Gastric Helicobacter pylori infection is not associated with alterations in gut microbiota composition in the large intestine. *Sci. Rep.* 11:13867. 10.1038/s41598-021-92836-734230584

[B24] Liou J. Lee Y. El-Omar E. Wu M. (2019). Efficacy and long-term safety of h. pylori eradication for gastric cancer prevention. *Cancers* 11:593. 10.3390/cancers11050593 31035365 PMC6562927

[B101] Liu Y. Liu J. Zhang H. Li M. Wang X. Chen J. (2022). Impact of Helicobacter pylori eradication therapy on the gut microbiota: A prospective cohort study. *Gut Microbes* 14:2038975. 10.1080/19490976.2022.2038975

[B25] Lopez-Siles M. Duncan S. Garcia-Gil L. Martinez-Medina M. (2017). Faecalibacterium prausnitzii: From microbiology to diagnostics and prognostics. *ISME J.* 11 841–852. 10.1038/ismej.2016.176 28045459 PMC5364359

[B26] Malfertheiner P. Megraud F. Rokkas T. Gisbert J. Liou J. Schulz C. (2022). Management of *Helicobacter* pylori infection: The Maastricht VI/Florence consensus report. *Gut* 10.1136/gutjnl-2022-327745 Online ahead of print. 35944925

[B27] Marshall B. Warren J. (1984). Unidentified curved bacilli in the stomach of patients with gastritis and peptic ulceration. *Lancet* 1 1311–1315. 10.1016/s0140-6736(84)91816-6 6145023

[B28] Mejías-Luque R. Gerhard M. (2020). Immune evasion strategies and persistence of *Helicobacter* pylori. *Curr. Top. Microbiol. Immunol.* 400 45–68. 10.1007/978-3-319-50520-6_3 28124149

[B29] Miquel S. Martín R. Rossi O. Bermúdez-Humarán L. Chatel J. Sokol H. (2013). Faecalibacterium prausnitzii and human intestinal health. *Curr. Opin. Microbiol.* 16 255–261. 10.1016/j.mib.2013.06.003 23831042

[B30] Młynarska E. Gadzinowska J. Tokarek J. Forycka J. Szponar-Łucińska K. Franczyk B. (2024). Exploring the significance of gut microbiota in diabetes pathogenesis and management-A narrative review. *Nutrients* 16:1938. 10.3390/nu16121938 38931292 PMC11206785

[B31] Morrison D. J. Preston T. (2016). Formation of short chain fatty acids by the gut microbiota and their impact on human metabolism. *Gut Microbes* 7 189–200. 10.1080/19490976.2015.1134082 26963409 PMC4939913

[B32] Myllyluoma E. Ahlroos T. Veijola L. Rautelin H. Tynkkynen S. Korpela R. (2007). Effects of anti-*Helicobacter* pylori treatment and probiotic supplementation on intestinal microbiota. *Int. J. Antimicrob. Agents* 29 66–72. 10.1016/j.ijantimicag.2006.08.034 17141481

[B33] Oh B. Kim B. Kim J. Kim J. Koh S. Kim B. (2016). The effect of probiotics on gut microbiota during the *Helicobacter* pylori eradication: Randomized controlled trial. *Helicobacter* 21 165–174. 10.1111/hel.12270 26395781

[B34] Raderer M. Kiesewetter B. (2020). How I treat MALT lymphoma: ‘a subjective interpretation of the gospel according to Isaacson’. *ESMO Open* 5:e000812. 10.1136/esmoopen-2020-000812 32723771 PMC7388885

[B35] Sugano K. Tack J. Kuipers E. Graham D. El-Omar E. Miura S. (2015). Kyoto global consensus report on *Helicobacter* pylori gastritis. *Gut* 64 1353–1367. 10.1136/gutjnl-2015-309252 26187502 PMC4552923

[B36] Treiber G. Lambert J. R. Weck M. N. (2003). Spontaneous disappearance of Helicobacter pylori antibodies in patients with advanced atrophic corpus gastritis. *APMIS 111*, 619–624. 10.1034/j.1600-0463.2003.1110604.x 12969017

[B37] Uemura N. Okamoto S. Yamamoto S. Matsumura N. Yamaguchi S. Yamakido M. (2001). *Helicobacter* pylori infection and the development of gastric cancer. *N. Engl. J. Med.* 345 784–789. 10.1056/NEJMoa001999 11556297

[B38] Wiklund A. Santoni G. Yan J. Radkiewicz C. Xie S. Birgisson H. (2025). Risk of gastric adenocarcinoma after eradication of *Helicobacter* pylori. *Gastroenterology* 169 244–250.e1. 10.1053/j.gastro.2025.01.239 39924057

[B39] Xu H. Zhang Y. Guo Y. Chen Y. Ju X. Guan X. (2024). Meta-analysis of the Correlation between Helicobacter Pylori Infection and the risk of Colorectal Neoplasia. (2024). *Altern. Ther. Health Med.* 30, 92–97. 10.1186/s12876-024-03323-7 38290458

[B40] You W. C. Zhang L. Gail M. H. Chang Y. S. Liu W. D. Ma J. L. (2000). Gastric dysplasia and gastric cancer: Helicobacter pylori, serum vitamin C, and other risk factors. *J. Natl. Cancer Inst*. 92, 1607–1612. 10.1093/jnci/92.19.1607 11018097

[B41] Zhao Y. Gao X. Guo J. Yu D. Xiao Y. Wang H. (2019). *Helicobacter* pylori infection alters gastric and tongue coating microbial communities. *Helicobacter* 24:e12567. 10.1111/hel.12567 30734438 PMC6593728

[B42] Zhou L. Lu H. Song Z. Lyu B. Chen Y. Wang J. (2022). 2022 Chinese national clinical practice guideline on Helicobacter pylori eradication treatment. *Chinese Med. J*. 135, 2899–2910. 10.1097/CM9.0000000000002546 36579940 PMC10106216

[B43] Zhu D. B. Wu J. M. (2019). Risk factors for treatment ineffectiveness in patients with H. pylori infection undergoing eradication treatment according to drug susceptibility testing results. *World Chinese J. Digestol.* 27 509–514. 10.11569/wcjd.v27.i8.509

